# Dosimetric comparison of proton therapy and CyberKnife in stereotactic body radiation therapy for liver cancers

**DOI:** 10.1007/s13246-024-01440-x

**Published:** 2024-05-29

**Authors:** Samuel Shyllon, Scott Penfold, Ray Dalfsen, Elsebe Kirkness, Ben Hug, Pejman Rowshanfarzad, Peter Devlin, Colin Tang, Hien Le, Peter Gorayski, Garry Grogan, Rachel Kearvell, Martin A Ebert

**Affiliations:** 1 School of Physics, Mathematics and Computing, The University of Western Australia, Perth, WA Australia; 2 Medical Technology and Physics, Sir Charles Gairdner Hospital, Nedlands, WA Australia; 3 Department of Radiation Oncology, Royal Adelaide Hospital, Adelaide, SA Australia; 4 Department of Physics, University of Adelaide, Adelaide, SA Australia; 5 Australian Bragg Centre for Proton Therapy and Research, Adelaide, SA Australia; 6 PT Product Engineering, Elekta, Adelaide, SA Australia; 7 Department of Radiation Oncology, Sir Charles Gairdner Hospital, Perth, WA Australia; 8 5D Clinics, Claremont, WA Australia; 9 Centre for Advanced Technologies in Cancer Research (CATCR), Perth, WA Australia; 10 Icon Cancer Centre, Midland, WA Australia; 11 Radiotherapy Physics, The Churchill Hospital, Headington, Oxford, UK; 12 GenesisCare, Adelaide, SA Australia

**Keywords:** Stereotactic radiotherapy, CyberKnife, Proton Therapy, Liver, Treatment planning

## Abstract

Stereotactic body radiation therapy (SBRT) has been increasingly used for the ablation of liver tumours. CyberKnife and proton beam therapy (PBT) are two advanced treatment technologies suitable to deliver SBRT with high dose conformity and steep dose gradients. However, there is very limited data comparing the dosimetric characteristics of CyberKnife to PBT for liver SBRT. PBT and CyberKnife plans were retrospectively generated using 4DCT datasets of ten patients who were previously treated for hepatocellular carcinoma (HCC, *N* = 5) and liver metastasis (*N* = 5). Dose volume histogram data was assessed and compared against selected criteria; given a dose prescription of 54 Gy in 3 fractions for liver metastases and 45 Gy in 3 fractions for HCC, with previously published consensus-based normal tissue dose constraints. Comparison of evaluation parameters showed a statistically significant difference for target volume coverage and liver, lungs and spinal cord (*p* < 0.05) dose, while chest wall and skin did not indicate a significant difference between the two modalities. A number of optimal normal tissue constraints was violated by both the CyberKnife and proton plans for the same patients due to proximity of tumour to chest wall. PBT resulted in greater organ sparing, the extent of which was mainly dependent on tumour location. Tumours located on the liver periphery experienced the largest increase in organ sparing. Organ sparing for CyberKnife was comparable with PBT for small target volumes.

**Supplementary Information:**

The online version contains supplementary material available at 10.1007/s13246-024-01440-x.

## Introduction

Liver cancer is the third most lethal cancer worldwide with approximately 0.8 million global deaths in 2020 [1]. The liver has the unique property of being a parallel organ (according to the critical volume model of radiobiology), therefore damage to a sufficiently small portion does not impair the function of the whole organ [2]. Consequently, suitable treatment modalities are limited to those that offer the highest level of conformality [3].

For patients with early stage hepatocellular carcinoma (HCC), surgical resection, percutaneous ablation techniques such as radiofrequency ablation (RFA) and microwave ablation (MWA) and liver transplantation are used with curative intent. For advanced cases, two common options are trans-arterial chemoembolization (TACE) and radiation therapy (internal and external) [4]. The use of external beam radiation therapy is achieved through the implementation of stereotactic body radiation therapy (SBRT), which ensures that high radiation doses are delivered to the tumour accurately, with steep dose gradients ensuring minimal dose to the surrounding critical structures in a few fractions. The nature of liver motion/deformation makes this task non-trivial, so that the successful implementation of SBRT must include motion management strategies such as active breathing control (ABC) (breath hold), abdominal compression, respiratory gating and real-time intra-fraction tumour tracking, which are commonly used in radiotherapy departments for a variety of moving targets [5–7].

These techniques allow the overall planning target volume (PTV) to be reduced. If target tracking or gating is not used, then the PTV must be based on an internal target volume (ITV) which accounts for all the possible locations that the tumour might occupy due to organ motion, mainly due to respiratory motion and the deformable nature of the liver [8].

The CyberKnife system (Accuray, Sunnyvale CA), is a platform that combines a compact Linear Accelerator that produces a 6 MV flattening filter free photon beam mounted on an image-guided robotic system to deliver SBRT, for the treatment of liver cancer as well as other critically-located targets [9–12]. The CyberKnife treatment system utilises the Accuray Synchrony® motion management system that employs an implanted fiducial marker as a surrogate to track the tumour accounting for respiratory motion - allowing minimisation of the GTV to PTV margin with no ITV required. The decrease in tumour target volume results in increased tumour prescription dose and an overall decrease in dose to normal tissues relative to other external beam photon techniques [8].

In addition to CyberKnife SBRT, proton beam therapy (PBT) has emerged as a suitable modality for the implementation of SBRT, which can be described as stereotactic body proton therapy (SBPT) [13]. The favourable depth-dose and off-axis profiles of the proton beam allows for increased dose escalation while keeping certain volumes of normal liver tissue free from receiving any more than extremely low doses [14]. The capability of real time tumour tracking has not yet been developed for PBT. Other motion compensation methods (e.g. ABC, voluntary breath hold, respiratory gating and abdominal compression) may be employed for the implementation of SBPT [15].

The CyberKnife system has already demonstrated efficacy and has been in clinical use for more than two decades to treat liver cancers [16–19], however, PBT using pencil beam scanning for liver targets is a relatively new technique. As a result, there is very limited data comparing the effectiveness of CyberKnife SBRT to PBT using pencil beam scanning. Therefore, the primary aim of this investigation was to perform a dosimetric comparison of CyberKnife SBRT and PBT for the treatment of HCC and liver metastases. The objective was a quantitative determination of modality superiority on a case per case level and the identification of treatment-specific factors that may favour one modality over the other. Economic considerations were not included.

## Methods

Ten patients who had previously been treated for HCC and liver metastasis (5 HCC and 5 liver metastases cases) and had 4DCT data available were selected for the study, approved under Sir Charles Gairdner Hospital Quality Improvement Activity 27,478.

The CyberKnife plans used a PTV that was based on the GTV contoured on the 50% breathing phase of the 4DCT (referred to as GTV50) plus a 5 mm setup uncertainty margin. The PBT plans used a PTV based on the ITV plus a 5 mm setup uncertainty margin. The ITV was generated from the sum of the GTV contoured on the 0% and 50% breathing phases, the GTV contoured on the Maximum Intensity Projection (MIP) dataset, and the GTV contoured on the free breathing scan. The GTV50 volumes ranged from 1.3 to 203.2 cm^3^ with a mean volume of 30.9 cm^3^ (SD=49.8). The ITV volumes ranged from 3.9 to 224.2 cm^3^ with a mean volume of 41.5 cm^3^ (SD = 55.2).

An additional PBT plan was generated for each patient based on PTV_GTV50_ to simulate the case where comparable motion management techniques were used. This is equivalent to the use of respiratory gating/breath hold for PBT. However, the nature of PBT using pencil beam scanning techniques could require a significant number of breath holds which may be difficult for patients to comply as discussed by Apisarnthanarax et al. (2017) [20]. Therefore, it may be more practical for some patients to be treated under free breathing conditions, so that the PBT is planned using the ITV. Hence the results for the PBT GTV50 plans are presented in the supplementary table and not discussed.

The CyberKnife plans were generated using the Accuray Precision v1.1 treatment planning software (TPS) with Monte Carlo dose calculation (Accuray Inc, Sunnyvale CA). Plans were generated using the CyberKnife Iris™ variable aperture using both the sequential and VOLO optimisation algorithm. Multileaf collimator was not used in the planning. Eclipse v13.7 TPS (Varian, Palo Alto CA) was used to generate the pencil beam scanning PBT plans with multi-field, robust optimisation using a relative biological effectiveness (RBE) of 1.1. Robust optimisation and evaluation was performed with a +/-5 mm geometric uncertainty along the principle patient dimensions (sup-inf, left-right, ant-post) and a 3% proton range uncertainty margin. The combination of geometric and range uncertainties resulted in 12 uncertainty scenarios. The proton pencil beam model utilised a spot size ranging from 8.5 to 11.7 mm FWHM in air at isocentre. A range shifter was used when required to deliver spots to superficial targets. A minimum 5 cm air gap between patient surface and range shifter was used. PBT plans included consideration of minimising skin dose by using multiple beams incident from different directions and by selecting beam directions that have a short target thickness in the beam direction. No consideration was given to treatment time during the planning process. The generated plans prescribed 54 Gy [RBE] in 3 fractions to the GTV for liver metastases and 45 Gy [RBE] in 3 fractions for HCC.

The UK consensus on normal tissue dose constraints for stereotactic radiotherapy were used [21]. The critical organs and their respective dose constraints are listed in Table 1.

**Table 1:** Summary of normal tissue dose constraints used to generate the treatment plans

Structure	Metric	Optimal	Mandatory	Label
Chest Wall	Dmax (0.5cm^3^)	< 37 Gy		Dmax < 37 Gy
	D30cm^3^	< 30 Gy		D30cc < 30 Gy
Combined Lung	V20 Gy		< 10%	V20Gy < 10%
Liver (Liver-GTV)– constraint for 5 fractions used	V10 Gy	< 70%		V10Gy < 70%
	Mean Dose	< 13 Gy	< 15.2 Gy	Mean Dose < 13 Gy
Skin	Dmax (0.5cm^3^)	< 33 Gy (Optimal);		Dmax < 33 Gy
	D10 cm^3^	< 30 Gy (Optimal)		D10cc < 30 Gy
Spinal Cord (canal)	Dmax (0.1cm^3^)	< 18 Gy	< 21.9 Gy	

D_max_ (0.1, 0.5 cm^3^) is the near-point maximum dose which is the minimum dose to the 0.1 cm^3^, 0.5 cm^3^ volume of the organ receiving the highest doses. D30 cm^3^ and D10 cm^3^ are the minimum doses received by respective volumes of 30 cm^3^ and 10 cm^3^ of the organ that gets the highest doses. V10Gy and V20Gy are the percentage volumes of the organs receiving doses of at least 10 Gy and 20 Gy, respectively [21].

The generated CyberKnife and PBT plans were then imported into 3D Slicer/Slicer RT [22], which was used to visualise the dose distribution and output the raw cumulative dose volume histogram (DVH) data for each plan in a comma separated value (.csv) format. MATLAB (2019a, Mathworks, Natick MA) was then used to apply formulations to generate the relevant target and normal tissue DVH metrics. To determine superiority, the non-parametric Wilcoxon signed rank test was used to establish the significance of any difference in metrics between the CyberKnife and PBT plans.

## Results

**Fig. 1:**
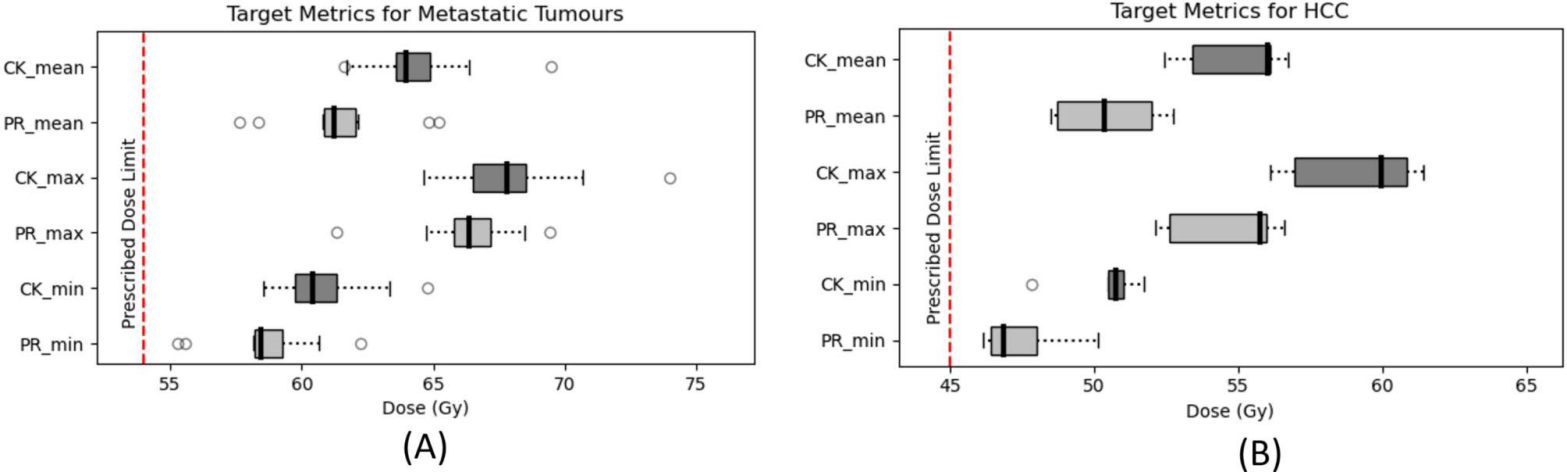
Box and whiskers plot of the minimum, maximum and mean dose to the target for CyberKnife (CK) and proton (PR) plans across the patients for the HCC **(A)** and metastatic cases **(B)** separately. The red dotted line on both plots indicates the prescribed doses which were 54 Gy and 45 Gy for the HCC and metastatic cases respectively. The black central mark on each box indicates the median, with the upper and lower of each box indicating the 25th and 75th percentiles. The outliers are indicated with circles and the whiskers extend to the most extreme data points not considered to be outliers

The planning goals/aims for the target were met for all patients for both CyberKnife and PBT plans based on the ITV + 5 mm. The difference between the PBT and CyberKnife minimum, maximum and mean doses to the target were significant (*p* < 0.05) with CyberKnife target doses being consistently higher than those of PBT plans for all patients as illustrated in Fig. 1.

**Fig. 2:**
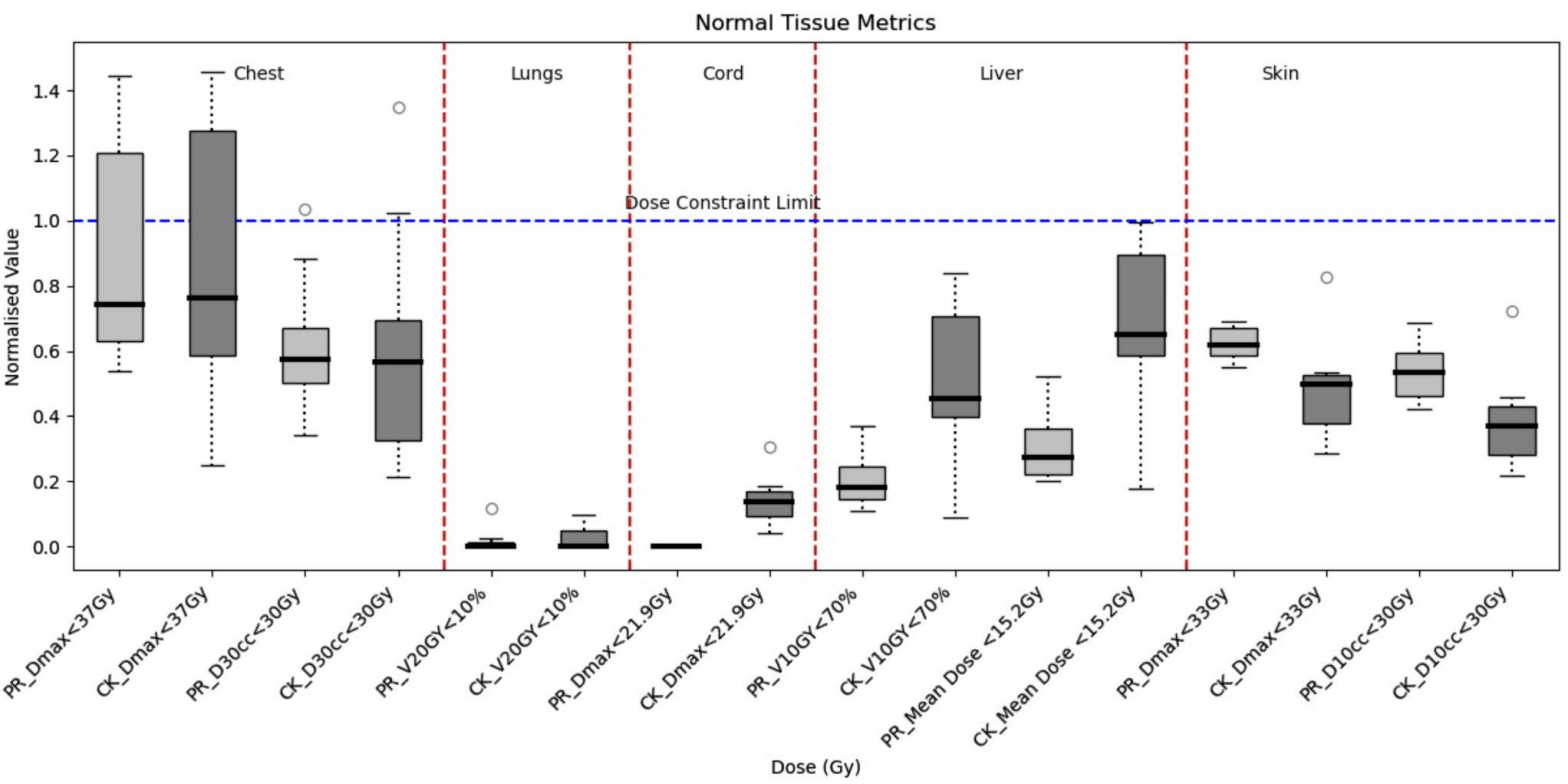
Box and whiskers plot of the normal tissue dose constraints listed in Table 1 for the liver, chest wall, skin, combined lungs, and the spinal cord. The black central mark on each box indicates the median, with the upper and lower of each box indicating the 25th and 75th percentiles. The outliers are indicated with circles and the whiskers extend to the most extreme data points not considered to be outliers. The resulting value of each metric was divided by the constraint limit to obtain a normalised value. Therefore, a value of 1 indicated by the blue dotted line represents the dose constraint limit

For the normal tissue constraints, all mandatory dose constraints were met for both CyberKnife and PBT plans as shown in Fig. 2.

Note that the normal liver dose metrics were based on the Liver minus GTV50 volume for both the CyberKnife and PBT plans. The CyberKnife plans did not meet/exceeded the optimal mean dose constraints to uninvolved liver (liver volume minus the target volume) for Patients 1, 5, 7 and 9. Patients 1 and 5 were HCC cases with a tumour volume of approximately 55.6 cm^3^ and 205 cm^3^, respectively. Patient 7 was a metastatic case with 2 target volumes of 3.4 cm^3^ and 47.3 cm^3^, while Patient 9 was a metastatic case with three target volumes of 7.2, 2.8 and 3.7 cm^3^. Patients 1, 5 and 7 had the three largest target volumes across all patients while patient 9 had one of the smallest target volumes (13.7 cm^2^ when combined) across all patients; however, two of the target volumes were in proximity to each other while the third target was relatively distant. As a result, two separate CyberKnife treatment plans were generated to treat two locations. The planned dose distributions were combined to make a composite dose distribution. Note that of all the metastatic cases (Patients 2, 7, 8, 9, 10), Patient 8 was the only other patient with target volumes that had a large degree of separation and two individual plans were needed. However, both target volumes were small (approximately 1.4 cm^3^), resulting in less dose to the uninvolved liver volume. Overall, analysis of the liver planning constraints, V10 Gy and mean dose to uninvolved liver (liver-GTV for both modalities) (see) indicated that the dose with PBT was significantly lower than the dose with CyberKnife (*p* < 0.05).

**Fig. 3:**
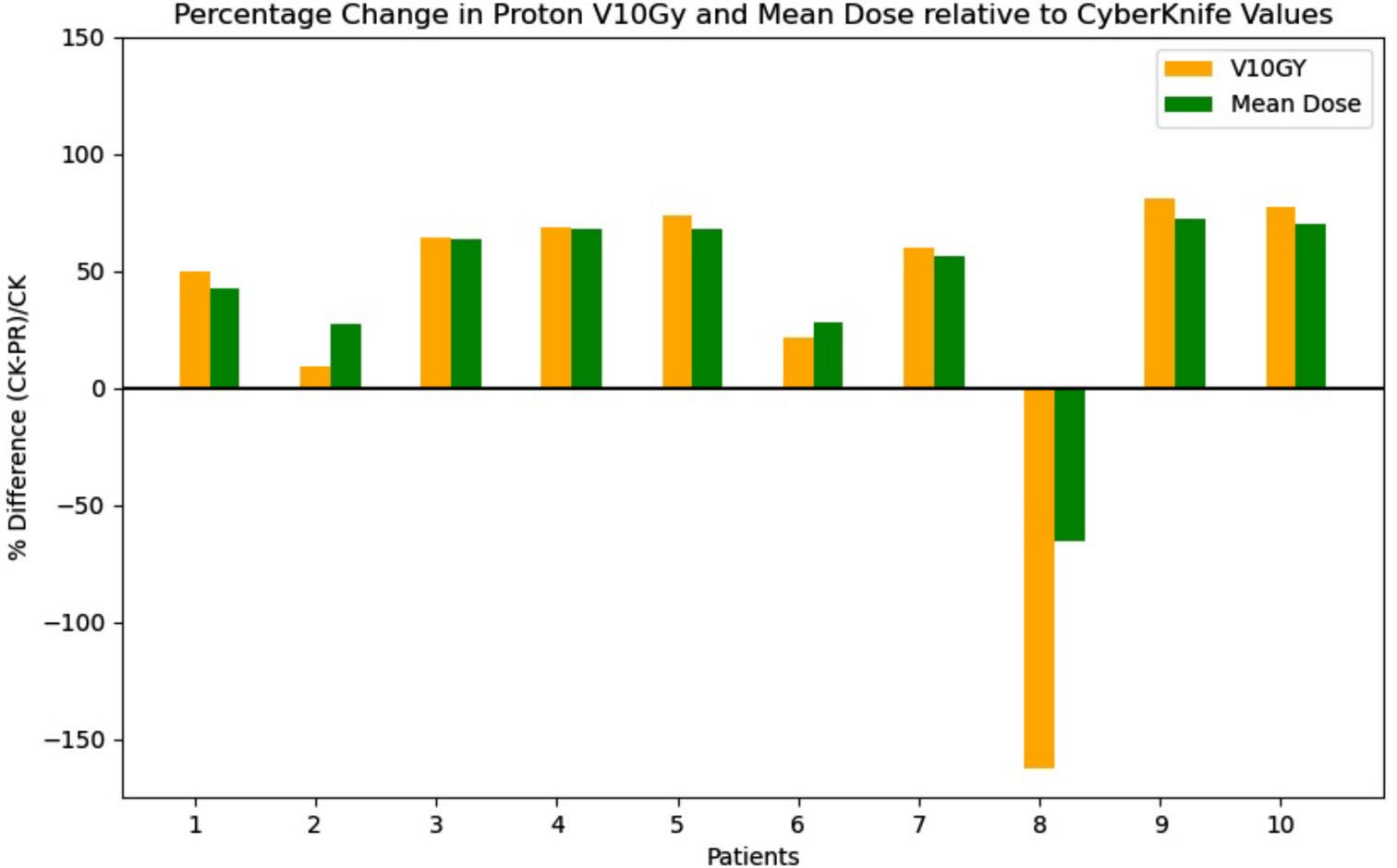
Plot of the percentage difference of the CyberKnife V10Gy and mean liver dose compared to respective values in the PBT plans

Significant difference was observed between the Dmax values in spinal cord using the CyberKnife and PBT (*p* < 0.05). This is due to the PBT plans resulting in 0 Gy Dmax to the spinal cord for all patients, while the CyberKnife plans resulted in doses greater than 0 Gy but significantly below the tolerance dose (21.9 Gy) with an average Dmax of 3.04 Gy.

The optimal constraints for the chest wall parameters (Dmax and D30cc) were not met for some plans using either of the modalities. Violation of the Dmax constraint (< 37 Gy) occurred in both plans for Patients 4, 5, 7 and 10, with Patient 4 being an HCC case with a volume of approximately 29 cm^3^, and Patient 10 being a metastatic case with 2 target volumes of 38.5 cm^3^ and 5.7 cm^3^. These patients also reported the highest mean dose to the target. Overall, there was no significant difference between the CyberKnife and the PBT plans across all the chest wall constraints (*p* > 0.05). The D30 cm^3^ (< 30 Gy) optimal constraint was only violated for the CyberKnife plan of Patient 5.

The differences between CyberKnife and PBT plans for the lung and skin constraints were not statistically significant (*p* > 0.05).

**Fig. 4:**
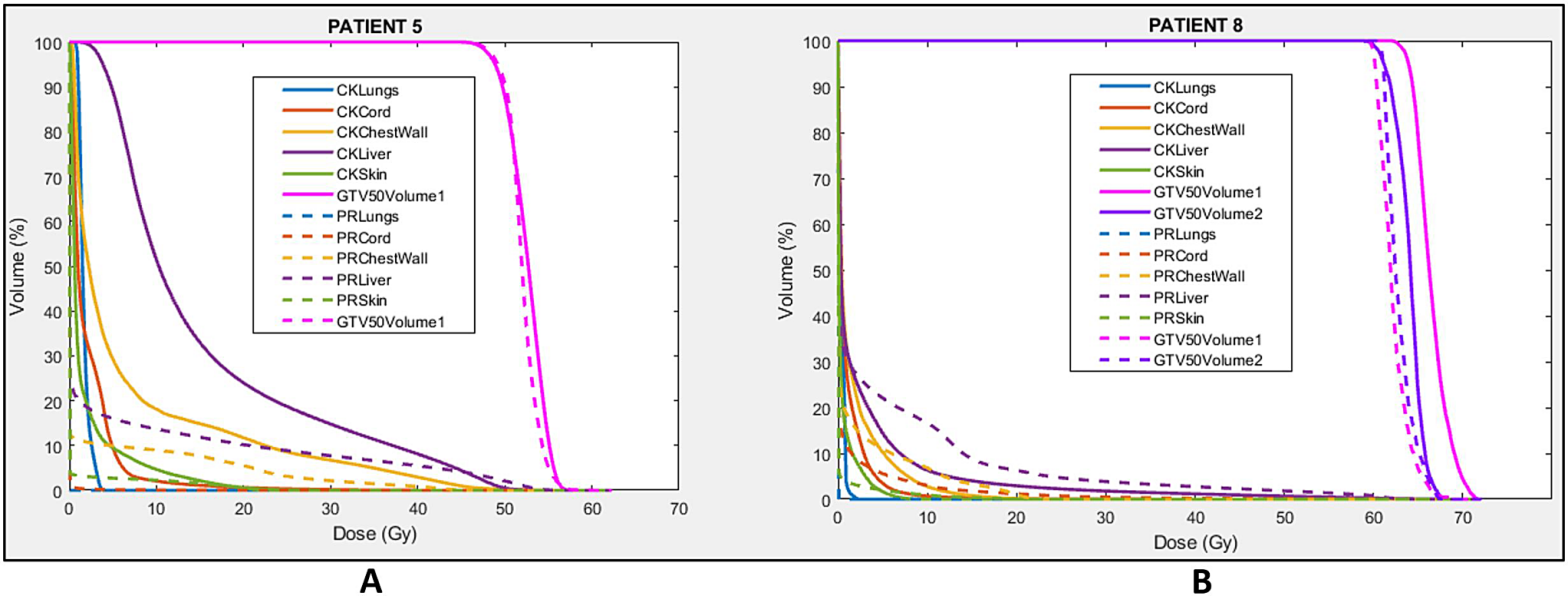
The DVH for the GTVs and normal tissues for patient 5 (A) and patient 8 (B). The CyberKnife plans are represented by the solid lines and PBT by the dashed lines. Patient 5 had a prescribed dose of 45 Gy and patient 8 had a prescribed dose of 54 Gy

With the exception of Patient 8, visual observation of the isodoses and DVH plots for all patients showed the PBT plans to have superior organ sparing. For patient 8, the CyberKnife plan displayed comparable organ sparing to the PBT. This is illustrated in Fig. 4. Figure 4A shows the DVH for Patient 5, which is representative of the DVH observed for the remaining nine patients. These results were due to Patient 8 having two lesions separated by some distance. This resulted in more of a low dose bath with PBT relative to CyberKnife than was required for all other patients. Furthermore, given the proximity of one of the Patient 8 target volumes to the liver-lung interface, 4 beams were utilised in Patient 8’s treatment plan to improve robustness and reduce the influence of any one beam.

**Fig. 5:**
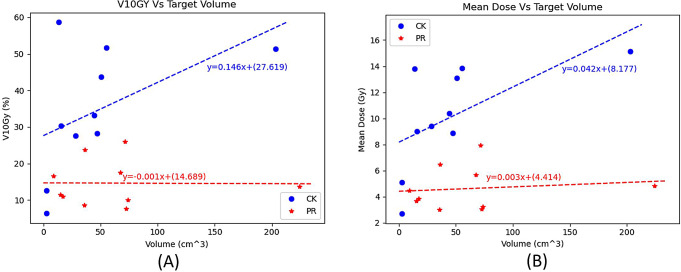
Plot of V10Gy (left) and mean dose to the liver (right) against target volume

For Patient 5 and 8 the percentage of the normal liver receiving greater than 0.5 Gy [RBE] for the PBT plans was 21.8% and 33.7%, while for the CyberKnife plans it was 94.8% and 41%, respectively. This shows that the amount of organ sparing is not heavily dependent on the size of the target for PBT, which is the opposite for the CyberKnife plans where organ sparing is increased for small target volumes. This characteristic is also demonstrated in Fig. 5, which shows that as target volume increases, the percentage of uninvolved liver volume receiving 10 Gy or more (V10Gy) and the mean dose to uninvolved liver increases for the CyberKnife plans (ranging from 6.33 to 58.7%) and remains relatively consistent on average for the PBT plans (ranging from 6.12 to 22.7%).

The percentage difference in V10Gy and mean uninvolved liver dose between the CyberKnife and PBT plans were computed and presented in Fig. 3.

**Fig. 6:**
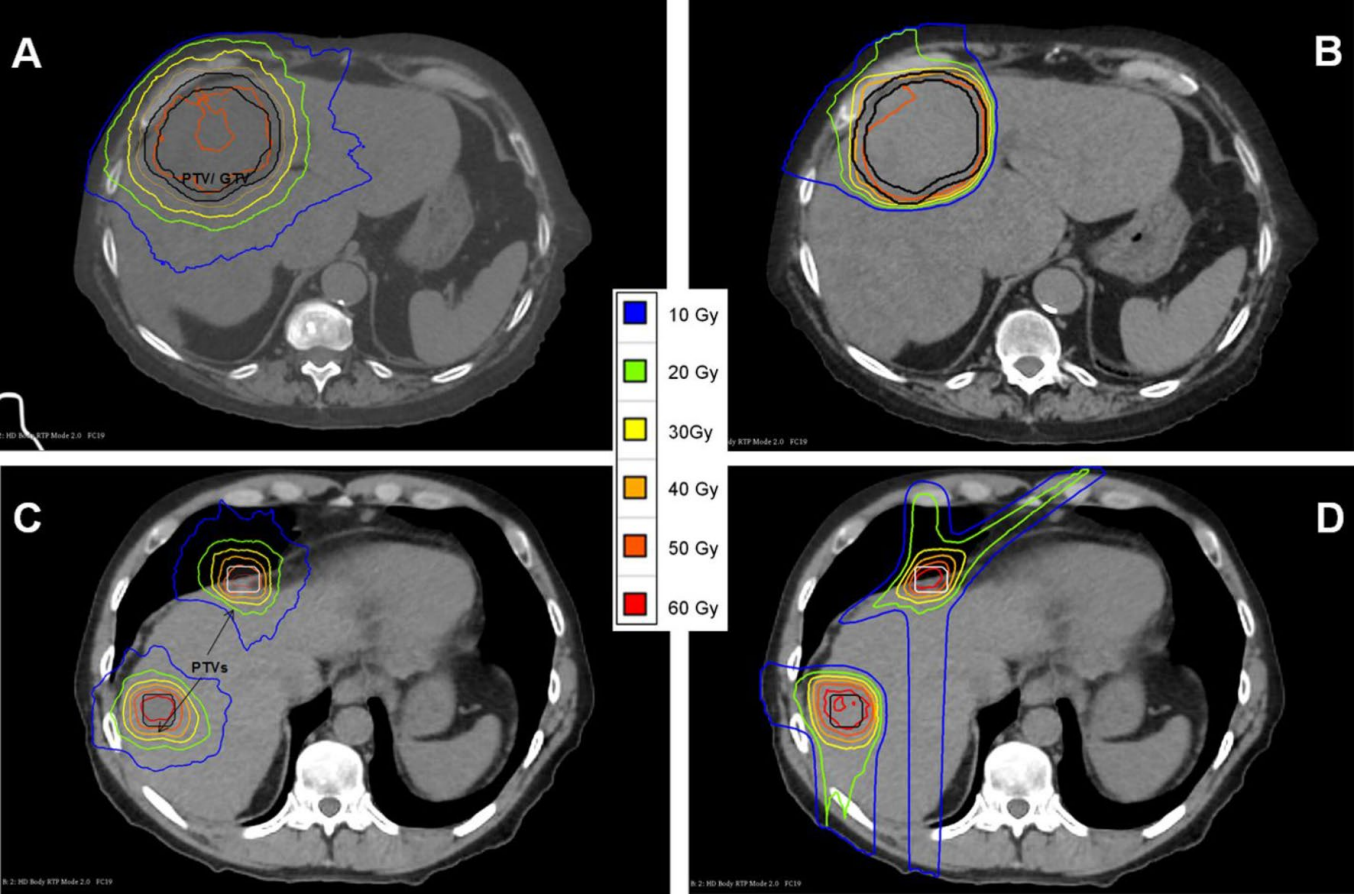
Dose distribution in the liver for Patients 5 and 8. **(A)** Patient 5 CyberKnife isodoses. **(B)** Patient 5 PBT isodoses. **(C)** Patient 8 CyberKnife isodoses. **(D)** Patient 8 PBT isodoses

Patients 3, 4, 5, 9 and 10 are observed to have much lower mean dose and irradiated volume in the PBT plans compared to the CyberKnife plans. All of these patients had targets located either on or close to the periphery of the liver and close to the chest wall, and the isodose representation showed that the proton beam was incident from directions such as the ones shown in Fig. 6C which minimises the amount of normal tissue traversed, resulting in the observed low doses to the uninvolved liver volume and pronounced differences compared to the CyberKnife plans.

## Discussion

In this study, a comparison was made between the CyberKnife and PBT treatment plans for primary and metastatic diseases of the liver. The results could help with the resource planning and clinical decision making for suitable patients once both therapeutic modalities become available to Australian patients in the future.

This study has compared PBT plans using an ITV to CyberKnife plans using a GTV; however, it should be noted that an ITV by nature includes a certain amount of normal tissue volume which would increase the risk of complications. To account for this, the same uninvolved liver volume (liver-GTV) was used in the analyses. Despite this, the findings were consistent with those of several others such as Petersen et al. (2011) who found that the PBT plans resulted in more organ sparing than photon-based plans [23]. The extent of the differences in organ sparing was shown by Arscott et al. (2017) and Apisarnthanarax et al. (2013) to be dependent on the tumour size and location in the liver [24, 25].

In the present study, the largest improvement in organ sparing of the normal liver was in cases with the targets appeared on the periphery of the liver and close to the chest wall. This indicates potential subsets of patients that may benefit the most from access to PBT. The study also showed that organ sparing for CyberKnife was comparable to the PBT plans for small target volumes, which suggests that patients with relatively small tumours may represent a subset of patients who should be given the least preference for PBT. These outcomes are significant since the current clinical care pathway for selection of PBT candidates in Australia requires that a photon-based plan be submitted (on a case by case basis) to the Medical Treatment Overseas Program (MTOP) which then submits the plan to the Royal Australian and New Zealand College of Radiologists (RANZCR) for review, after which the patient may be financially supported for overseas treatment [26]. With Australia’s first PBT facility currently in development, PBT will be more accessible to Australian patients negating the need for overseas treatment [27]. Therefore, there is need for the establishment of national protocols to determine the superiority of PBT over other available treatment modalities to ensure that patients who will truly benefit from PBT are referred. As such, the subsets of patients identified in the study that are likely to benefit from PBT can potentially be incorporated into such a protocol. However, optimal protocols incorporate factors such as the treatment time for the chosen treatment modality, patient comfort, accessibility of the treatment facility and cost of treatment. Indeed, the radiation oncology literature is not new to other examples where a cheap and easy-to-access treatment option is as effective as one characterized by high-burden costs for health services [28, 29].

The outcome of this study showing PBT to be superior from a dosimetric point of view despite the use of a larger volume (ITV) may appear unintuitive. However, this is a consequence of liver being the main organ of concern. The liver is a relatively large organ and the target sizes are small relative to it; thus, the decreased margins for the CyberKnife do not correspond to a dosimetric advantage.

Motion management is an important consideration in pencil beam scanning for mobile targets, such as the liver. Strategies such as repainting, gating, and re-gating have been proposed for managing motion in pencil beam scanning delivery. Due to the longer beam-on times commonly encountered in proton therapy compared to X-ray therapy, this work primarily focuses on non-gated delivery. In this context, repainting is a potential method to reduce the interplay effects associated with pencil beam scanning. It’s important to note that repainting does not alter the dosimetry presented in the treatment planning system and was not explicitly included in this work. However, for small targets with brief beam-on times, gating may also serve as a viable alternative.

In accordance with the International Commission of Radiation Units and Measurements (ICRU) report 78 (2007), this study has assumed an RBE of 1.1, which implies that protons are assumed to be 10% more biologically damaging than photons per unit dose. This assumption is being challenged due to evidence that the RBE changes with depth into the tissue. This reality means that proton dose distributions generated by the PBT plans are theoretical and may change depending on the way in which the RBE is accounted for. Lowe et al. (2020) have developed guidelines for comparing PBT plans to photon plans for clinical trials and highlights the potential effect of a variable RBE and it’s consideration in the planning process [30]. Several publications have proposed the use of a variable RBE to calculate the dose distribution such as that by Jones, McMahony & Prise (2018) and Chen et al. (2018) [15, 31]. If such methods are used, it could lead to significant changes in the observed dose distribution within the target.

Given the limited literature comparing CyberKnife to PBT, the authors’ aspiration is that this study will establish a foundation for future research. This was a limited retrospective study generating hypotheses to guide more extensive work. In addition to the presented conventional treatment plan comparison parameters, additional analyses were performed incorporating conformity indices, homogeneity indices and radiobiological models. However, these additional analyses did not provide any further insights over and above those presented here. It is recommended that in larger, subsequent investigations, such additional analyses would provide more robust measures for distinguishing the relative merits of each treatment modality.

Note that the decision to limit the study to 10 cases was mainly due to the availability of clinicians to delineate and generate the treatment plans within the timeframe that the authors were constrained to. Furthermore, this was considered sufficient to see the primary patterns, aligning with similar studies conducted by Petersen et al. (2011), Arscott et al. (2017) and Apisarnthanarax et al. (2013) [15, 16, 23]. However, it is evident that a larger dataset would provide further insights such as the derivation of a potential cut-off tumour size (or tumour size to liver size ratio) where PBT could exhibit a significant advantage over CyberKnife, and vice versa. Additionally, a larger dataset would facilitate a comparison of the magnitude of PBT dosimetric benefit between tumours in the left and right lobes of the liver. These parameters would allow for greater segregation of patients for whom PBT would offer superior dosimetric advantage. The authors express their aspiration to acquire more data for analysis and to present the results in future studies.

CyberKnife plans consistently demonstrate elevated minimum, maximum, and mean dose values delivered to targets. This is primarily because CyberKnife treatments do not adhere to the International Commission on Radiation Units and Measurements (ICRU) guidelines. The ICRU recommends delivering a minimum dose of 95% to a maximum dose of 107% within the target area and prioritizes a homogeneous dose distribution. Typically, CyberKnife treatment plans prescribe doses at isodose lines ranging from 70 to 80%, making it common to observe maximum doses up to 140% of the prescribed dose within the target, with a focus on ablation [23, 31]. Additionally, the difference in the physics of CyberKnife and protons may contribute to the observed differences, particularly in the shape of the penumbra. Steeper penumbras typically mean that a higher prescription isodose will be required to ensure adequate coverage is achieved.

Considering that CyberKnife plans are currently the benchmark for clinical acceptability there arises an opportunity for dose escalation in PBT plans. To explore this possibility, the doses in PBT plan doses are scaled to match those of the CyberKnife plans. This scaling was done using the Dose Accumulation feature of Slicer RT for Proton GTV plans across all 10 patients, employing a scaling factor based on the mean dose comparison between Proton GTV and CyberKnife plans.

Post-scaling, the difference in target Dose-Volume Histogram (DVH) metrics between CyberKnife and scaled PBT plans was not significant, evidenced by a p-value greater than 0.05 for the mean, minimum, and maximum dose to the target. The evaluation and comparison of normal tissue plans for the scaled proton plan against the CyberKnife yielded similar discussions and conclusions as with the unscaled plans, albeit with minor variations in numerical values. The results for the scaled GTV plans are detailed in the supplementary table.

Future research endeavours should extend to a comparative analysis of state-of-the-art linear accelerator plans against PBT, particularly focusing on ‘larger’ target volumes, which are not ideal for CyberKnife treatment. Evaluating the efficacy and organ sparing of these methods could inform optimal strategies for larger targets, addressing a key gap in the literature and aiding in treatment decisions.

## Conclusion

This study made a comparison of planned doses for CyberKnife SBRT and PBT for the treatment of HCC and liver metastases. The proton plans resulted in more sparing of organs at risk compared to the CyberKnife plans, with the extent of difference in organ sparing depending mainly on the tumour size and location within the liver. This study identified a subset of patients that may benefit the most from PBT to be those with targets located towards the periphery of the liver. It also identified a subset of patients that will benefit the least to be those with relatively small targets.

## Electronic supplementary material

Below is the link to the electronic supplementary material.

Supplementary Material 1

## Data Availability

Data cannot be shared due to ethical, and legal restrictions.
