# Supplementary material for: Dosimetric comparison of proton therapy and CyberKnife in stereotactic body radiation therapy for liver cancers
Source: Phys Eng Sci Med. 2024 May 29;47(3):1203–12. doi: 10.1007/s13246-024-01440-x (PMC11408538; doi:10.1007/s13246-024-01440-x)
Supplement: Supplementary file 1 — Supplementary Material 1 [file 13246_2024_1440_MOESM1_ESM.docx]

**Supplementary Material**

The tables below show the calculated DVH metrics for planning constraints for the target volumes and normal tissue structures. The yellow shading indicates where the optimal dose constraints have been violated and the red shading indicates where the mandatory dose constraints have been violated. PR_GTV refers to the proton plans based on the GTV, PR_ITV refers to the plans based on the ITV and CK_GTV refers to the CyberKnife plans which are based on the GTV. GTV2/3 are for the metastatic cases which have multiple target volumes within the liver. Note that patient 2 a metastatic case had 2 target volumes, however they were in close proximity to one so that it was planned using one target volume that encompassed both.

Table A1: Summary of calculated DVH metrics for planning constraints for each target volumes.

| **PROTON GTV1** | | | | **CK GTV1** | | | | **PROTON ITV1** | | | |
| --- | --- | --- | --- | --- | --- | --- | --- | --- | --- | --- | --- |
| Volume (cc) | meandose (GY) | maxdose (GY) | mindose(GY) | Volume (cc) | meandose (GY) | maxdose (GY) | mindose(GY) | Volume (cc) | meandose (GY) | maxdose (GY) | mindose(GY) |
| 38.5 | 57.2 | 63.3 | 55.6 | 38.6 | 64.3 | 68.6 | 60.4 | 61.8 | 57.6 | 61.3 | 55.3 |
| 55.7 | 48.4 | 52.3 | 46.3 | 55.4 | 56.0 | 60.0 | 51.7 | 71.6 | 48.5 | 52.1 | 46.2 |
| 3.3 | 63.2 | 65.1 | 60.7 | 2.8 | 69.5 | 74.0 | 64.8 | 15.1 | 65.2 | 68.5 | 62.2 |
| 48.0 | 50.5 | 54.5 | 48.8 | 47.2 | 56.1 | 60.9 | 50.7 | 73.9 | 48.7 | 52.6 | 46.4 |
| 30.2 | 49.2 | 56.8 | 46.6 | 28.3 | 56.7 | 61.4 | 51.0 | 35.6 | 50.4 | 56.6 | 46.9 |
| 205.8 | 49.9 | 53.6 | 47.4 | 203.2 | 52.4 | 56.1 | 47.8 | 224.2 | 52.0 | 56.0 | 48.0 |
| 15.4 | 51.7 | 55.7 | 49.3 | 15.6 | 53.4 | 57.0 | 50.5 | 36.1 | 52.7 | 55.7 | 50.1 |
| 3.3 | 61.6 | 67.5 | 57.7 | 3.5 | 61.6 | 65.1 | 58.5 | 6.5 | 64.8 | 69.4 | 60.7 |
| 1.7 | 62.8 | 67.0 | 59.3 | 1.4 | 66.4 | 70.7 | 63.3 | 4.7 | 61.7 | 66.4 | 59.4 |
| 7.2 | 60.5 | 67.1 | 58.6 | 7.2 | 63.7 | 67.6 | 60.2 | 8.3 | 61.1 | 66.4 | 58.5 |
|  |  |  |  |  |  |  |  |  |  |  |  |
| **PROTON GTV2** | | | | **CK GTV2** | | | | **PROTON ITV2** | | | |
| Volume (cc) | meandose (GY) | maxdose (GY) | mindose(GY) | Volume (cc) | meandose (GY) | maxdose (GY) | mindose(GY) | Volume (cc) | meandose (GY) | maxdose (GY) | mindose(GY) |
| 5.6 | 58.2 | 64.5 | 56.5 | 5.8 | 64.0 | 68.0 | 59.6 | 10.8 | 58.4 | 64.8 | 55.6 |
| 47.3 | 59.2 | 63.1 | 56.6 | 47.3 | 63.6 | 66.3 | 60.4 | 60.9 | 62.1 | 67.4 | 58.4 |
| 1.3 | 62.4 | 66.0 | 59.2 | 1.2 | 63.9 | 67.0 | 60.5 | 4.3 | 61.3 | 65.7 | 59.0 |
| 2.8 | 60.9 | 66.1 | 58.7 | 2.8 | 61.7 | 64.6 | 59.0 | 4.8 | 60.8 | 66.4 | 58.5 |
|  |  |  |  |  |  |  |  |  |  |  |  |
| **PROTON GTV3** | | | | **CK GTV3** | | | | **PROTON ITV3** | | | |
| Volume (cc) | meandose (GY) | maxdose (GY) | mindose(GY) | Volume (cc) | meandose (GY) | maxdose (GY) | mindose(GY) | Volume (cc) | meandose (GY) | maxdose (GY) | mindose(GY) |
| 3.7 | 61.6 | 69.4 | 58.8 | 3.7 | 65.1 | 68.2711 | 61.6401 | 3.9 | 61.0 | 66.1 | 58.1 |

Table A2: Comparison of Proton GTV, ITV and CK planning constraints for target volumes .

|  | **Mean value** | | | **P-Value** | |
| --- | --- | --- | --- | --- | --- |
| **Parameter** | **CK** | **PR_GTV** | **PR_ITV** | **CK-PR_GTV** | **CK-PR_ITV** |
| Volume | 30.9 | 31.3 | 41.5 | 0.09 | 0.0001 |
| MeanDose | 61.2 | 57.1 | 57.8 | 0.0001 | 0.001 |
| Max Dose | 65.0 | 62.1 | 62.4 | 0.01 | 0.01 |
| Min Dose | 57.3 | 54.7 | 54.9 | 0.0001 | 0.002 |

Table A3: Summary of calculated DVH metrics for planning constraints for normal tissue structures

| **LIVER** | | | | | | | |
| --- | --- | --- | --- | --- | --- | --- | --- |
|  |  | PR GTV | CK | PR ITV | PR GTV | CK | PR ITV |
| patient # | Tumour Type | V10GY<70% | V10GY<70% | V10GY<70% | Mean Dose<13Gy | Mean Dose<13Gy | Mean Dose<13Gy |
| 10 | METs | 6.12 | 33.25 | 6.9058 | 2.32 | 10.40 | 2.6671 |
| 1 | HCC | 22.68 | 51.64 | 24.66 | 6.65 | 13.86 | 7.28 |
| 2 | METs | 6.61 | 12.65 | 10.94 | 1.98 | 5.10 | 3.33 |
| 3 | HCC | 7.47 | 28.20 | 8.98 | 2.29 | 8.86 | 2.71 |
| 4 | HCC | 8.34 | 27.61 | 8.23 | 2.85 | 9.42 | 2.81 |
| 5 | HCC | 13.55 | 51.22 | 22.84 | 4.65 | 15.12 | 5.91 |
| 6 | HCC | 17.47 | 30.24 | 16.89 | 4.61 | 8.99 | 5.28 |
| 7 | METs | 18.48 | 43.75 | 16.33 | 5.89 | 13.08 | 4.27 |
| 8 | METs | 8.94 | 6.33 | 10.91 | 2.43 | 2.70 | 3.74 |
| 9 | METs | 11.58 | 58.68 | 10.91 | 3.94 | 13.81 | 3.74 |
|  |  |  |  |  |  |  |  |
| **CHEST** | | | | | | | |
|  |  | Pr GTV | CK | PR ITV | Pr GTV | CK | PR ITV |
| patient # | Tumour Type | Dmax<37Gy | Dmax<37Gy | Dmax<39Gy | D30cc<30Gy | D30cc<30Gy | D30cc<30Gy |
| 10 | METs | 53.4 | 48.19 | 53.4 | 24.24 | 30.76 | 31.0 |
| 1 | HCC | 24.2 | 22.40 | 24.2 | 17.43 | 15.32 | 18.6 |
| 2 | METs | 23.3 | 9.85 | 23.3 | 6.23 | 6.54 | 16.6 |
| 3 | HCC | 30.8 | 33.07 | 30.8 | 16.93 | 20.91 | 17.7 |
| 4 | HCC | 48.3 | 50.02 | 48.3 | 16.46 | 18.72 | 16.6 |
| 5 | HCC | 45.0 | 47.80 | 45.0 | 27.96 | 38.69 | 26.4 |
| 6 | HCC | 19.8 | 9.15 | 19.8 | 13.07 | 6.40 | 14.5 |
| 7 | METs | 43.4 | 44.14 | 43.4 | 21.21 | 20.80 | 20.7 |
| 8 | METs | 22.5 | 21.36 | 22.5 | 8.30 | 8.15 | 12.9 |
| 9 | METs | 23.6 | 23.25 | 23.6 | 10.81 | 14.68 | 10.2 |
|  |  |  |  |  |  |  |  |
| **SKIN** | | | | | | | |
|  |  | Pr GTV | CK | PR ITV | Pr GTV | CK | PR ITV |
| patient # | Tumour Type | Dmax<33Gy | Dmax<33Gy | Dmax<33Gy | D10cc<21.9Gy | D10cc<30Gy | D10cc<30Gy |
| 10 | METs | 21.44 | 17.60 | 22.5 | 17.97 | 12.84 | 19.5 |
| 1 | HCC | 20.05 | 12.42 | 18.4 | 16.91 | 10.03 | 11.4 |
| 2 | METs | 19.73 | 12.64 | 20.6 | 8.08 | 8.47 | 15.0 |
| 3 | HCC | 20.97 | 17.32 | 19.5 | 16.94 | 13.70 | 16.8 |
| 4 | HCC | 17.87 | 15.81 | 18.9 | 15.88 | 9.87 | 15.9 |
| 5 | HCC | 22.94 | 27.29 | 22.5 | 20.12 | 21.64 | 20.6 |
| 6 | HCC | 17.52 | 12.39 | 18.1 | 11.87 | 6.87 | 13.5 |
| 7 | METs | 22.44 | 17.41 | 22.8 | 18.03 | 12.24 | 18.2 |
| 8 | METs | 24.55 | 9.37 | 20.2 | 9.29 | 6.57 | 13.2 |
| 9 | METs | 20.81 | 17.13 | 21.0 | 16.08 | 12.88 | 16.2 |
|  |  |  |  |  |  |  |  |
|  |  | **LUNG** | | | **CORD** | | |
|  |  | Pr GTV | CK | PR ITV | Pr GTV | CK | PR ITV |
| patient # | Tumour Type | V20GY<10% | V20GY<10% | V20GY<10% | Dmax<21.9Gy | Dmax<21.9Gy | Dmax<21.9Gy |
| 10 | METs | 0.17 | 0.97 | 0.2 | 0.00 | 1.13 | 0.00 |
| 1 | HCC | 0.00 | 0.00 | 0.0 | 0.00 | 3.74 | 0.00 |
| 2 | METs | 0.00 | 0.00 | 0.0 | 0.00 | 3.53 | 0.00 |
| 3 | HCC | 0.16 | 0.92 | 0.2 | 0.00 | 4.08 | 0.00 |
| 4 | HCC | 0.00 | 0.00 | 0.0 | 0.00 | 2.54 | 0.00 |
| 5 | HCC | 0.01 | 0.63 | 0.0 | 0.00 | 3.67 | 0.00 |
| 6 | HCC | 0.00 | 0.00 | 0.0 | 0.00 | 0.90 | 0.00 |
| 7 | METs | 0.00 | 0.00 | 1.2 | 0.00 | 2.09 | 0.00 |
| 8 | METs | 0.89 | 0.13 | 0.0 | 0.00 | 2.05 | 0.00 |
| 9 | METs | 0.00 | 0.01 | 0.0 | 0.00 | 6.68 | 0.00 |

Table A4: Comparison of Proton GTV, ITV and CK planning constraints for normal tissue structures.

|  |  | **Mean value** | | | **P-Value** | | |
| --- | --- | --- | --- | --- | --- | --- | --- |
| **Structure** | **Parameter** | **CK** | **PR_GTV** | **PR_ITV** | **CK-PR_GTV** | **CK-PR_ITV** | **PR_GTV--PR_ITV** |
| **LIVER** | V10Gy liver | 34.4 | 12.1 | 14 | 0.004 | 0.01 | 0.083984 |
|  | Mean Dose liver | 10.1 | 3.8 | 4.3 | 0.002 | 0.004 | 0.064453 |
| **CHEST WALL** | Dmax chest | 30.9 | 32.5 | 33.4 | 0.922 | 0.432 | 0.16016 |
|  | D30cc chest | 18.1 | 16.3 | 18.5 | 0.322 | 0.77 | 0.13086 |
| **SKIN** | Dmax skin | 20.7 | 24.2 | 24 | 0.16 | 0.131 | 0.8457 |
|  | D10cc skin | 16.3 | 18.5 | 19.9 | 0.16 | 0.105 | 0.13086 |
| **CORD** | Dmax cord | 3 | 0 | 0 | 0.002 | 0.002 | 1 |
| **LUNGS** | V20 Gy | 0.3 | 0.1 | 0.2 | 0.313 | 0.625 | 0.125 |

**Scaled Proton Plan Results**

Table B1: Summary of calculated DVH metrics for planning constraints for each target volumes.

| **SCALE PROTON DOSE** | | | | | | | | | | | | | |
| --- | --- | --- | --- | --- | --- | --- | --- | --- | --- | --- | --- | --- | --- |
| **PROTON GTV1** | | | |  | **PROTON GTV2** | | | |  | **PROTON GTV3** | | | |
| Volume (cc) | mean (GY) | max (GY) | min (GY) |  | Volume (cc) | mean (GY) | max (GY) | min (GY) |  | Volume (cc) | mean (GY) | max (GY) | min (GY) |
| 38.5 | 58.3 | 64.6 | 56.7 |  | 5.6 | 59.4 | 65.7 | 57.6 |  | 3.7 | 61.7 | 69.5 | 58.9 |
| 55.7 | 56 | 60.6 | 53.6 |  | 47.3 | 61.4 | 65.4 | 58.7 |  |  |  |  |  |
| 3.3 | 69.5 | 71.5 | 66.7 |  | 1.3 | 62.4 | 66 | 59.2 |  |  |  |  |  |
| 48 | 56.1 | 60.6 | 54.2 |  | 2.8 | 61 | 66.1 | 58.8 |  |  |  |  |  |
| 30.2 | 56.8 | 65.5 | 53.7 |  |  |  |  |  |  |  |  |  |  |
| 205.8 | 52.4 | 56.2 | 49.7 |  |  |  |  |  |  |  |  |  |  |
| 15.4 | 53.4 | 57.5 | 50.9 |  |  |  |  |  |  |  |  |  |  |
| 3.3 | 63.9 | 70.1 | 59.9 |  |  |  |  |  |  |  |  |  |  |
| 1.7 | 62.8 | 67 | 59.3 |  |  |  |  |  |  |  |  |  |  |
| 7.2 | 60.6 | 67.3 | 58.7 |  |  |  |  |  |  |  |  |  |  |

Table B2: Comparison of Proton GTV, ITV and CK planning constraints for target volumes .

|  | **Mean value** | | **P-Value** |
| --- | --- | --- | --- |
| **Parameter** | **CK** | **PR_GTV** | **CK-PR_GTV** |
| Volume | 30.9 | 31.3 | 0.3 |
| MeanDose | 61.2 | 59.7 | 0.7 |
| Max Dose | 65.0 | 64.9 | 0.1 |
| Min Dose | 57.3 | 57.1 | 0.2 |

Table B3: Summary of calculated DVH metrics for planning constraints for normal tissue structures

|  | | **LIVER** | |  | **CHEST** | |  | **SKIN** | |  | **LUNG** |  | **CORD** |
| --- | --- | --- | --- | --- | --- | --- | --- | --- | --- | --- | --- | --- | --- |
|  |  | PR GTV | PR GTV |  | Pr GTV | Pr GTV |  | Pr GTV | Pr GTV |  | Pr GTV |  | Pr GTV |
| patient # | Tumour Type | V10GY<70% | Mean Dose<13Gy |  | Dmax<37Gy | D30cc<30Gy |  | Dmax<33Gy | D10cc<21.9Gy |  | V20GY<10% |  | Dmax<21.9Gy |
| 10 | METs | 6.17 | 2.32 |  | 54.36 | 24.24 |  | 23.82 | 17.97 |  | 0.21 |  | 0.00 |
| 1 | HCC | 23.75 | 6.65 |  | 25.03 | 17.43 |  | 23.21 | 16.91 |  | 0.00 |  | 0.00 |
| 2 | METs | 6.91 | 1.98 |  | 26.12 | 6.23 |  | 21.68 | 8.08 |  | 0.00 |  | 0.00 |
| 3 | HCC | 7.77 | 2.29 |  | 28.69 | 16.93 |  | 23.31 | 16.94 |  | 0.19 |  | 0.00 |
| 4 | HCC | 8.74 | 2.85 |  | 54.99 | 16.46 |  | 20.60 | 15.88 |  | 0.00 |  | 0.00 |
| 5 | HCC | 13.74 | 4.65 |  | 46.99 | 27.96 |  | 23.39 | 20.12 |  | 0.01 |  | 0.00 |
| 6 | HCC | 17.75 | 4.61 |  | 20.01 | 13.07 |  | 18.11 | 11.87 |  | 0.00 |  | 0.00 |
| 7 | METs | 18.65 | 5.89 |  | 42.92 | 21.21 |  | 23.26 | 18.03 |  | 0.00 |  | 0.00 |
| 8 | METs | 8.94 | 2.43 |  | 29.08 | 8.30 |  | 25.55 | 9.29 |  | 0.96 |  | 0.00 |
| 9 | METs | 11.58 | 3.94 |  | 24.00 | 10.81 |  | 21.25 | 16.08 |  | 0.00 |  | 0.00 |

Table B4: Comparison of Proton GTV, ITV and CK planning constraints for normal tissue structures.

|  |  | **Mean value** | | **P-Value** |
| --- | --- | --- | --- | --- |
| **Structure** | **Parameter** | **CK** | **PR_GTV** | **CK-PR_GTV** |
| **LIVER** | V10Gy liver | 34.4 | 12.4 | 0.004 |
|  | Mean Dose liver | 10.1 | 4 | 0.002 |
| **CHEST WALL** | Dmax chest | 30.9 | 35.2 | 0.084 |
|  | D30cc chest | 18.1 | 17.5 | 1.000 |
| **SKIN** | Dmax skin | 20.7 | 22.4 | 0.131 |
|  | D10cc skin | 16.3 | 16.3 | 0.131 |
| **CORD** | Dmax cord | 3 | 0 | 0.002 |
| **LUNGS** | V20 Gy | 0.3 | 0.1 | 0.004 |
